# Meta-analysis of reported presacral myelolipomas, including a report of a new case

**DOI:** 10.1186/s13256-022-03746-4

**Published:** 2023-02-01

**Authors:** Congde Xu, Atsuko Kasajima, Alexander Novotny, Helmut Friess

**Affiliations:** 1 Klinikum rechts der Isar, Klinik und Poliklinik für Chirurgie, TU München, Ismaninger Straße 22, 81675 Munich, Deutschland; 2 Klinikum rechts der Isar, Institut für Allgemeine Pathologie und Pathologische Anatomie, TU München, Ismaninger Straße 22, 81675 Munich, Deutschland

**Keywords:** Presacral myelolipoma, Diagnostic, Treatment, Meta-analysis

## Abstract

**Background:**

Presacral myelolipomas form a rare disease and are often found incidentally in imaging diagnostics.

**Case presentation:**

In this study, we report the case of a 71-year-old caucasian female with an incidental finding of a retroperitoneal tumor on magnetic resonance imaging scan. This report aimed at presenting the clinical course of this patient with emphasis on analysis of pathological, clinical, and epidemiological features in a meta-analysis of reported cases.

**Conclusion:**

Presacral myelolipomas are rare and its etiology remains unclear. Surgical resection is indicated in symptomatic lesions and lesions > 4 cm. More clinical and pathological research on this rare entity is warranted.

## Background

Myelolipomas are benign tumors that are composed of mature adipose tissue and elements of extramedullary hematopoiesis with trilinear hematopoietic cells [1] with unknown etiology.

They were first described by Gierke in 1909 [2] and named by Oberling in 1929 [3]. Most commonly, myelolipomas are found as incidentalomas in adrenal glands [4]. Incidence ranges from 1:500 to 1:2500 in autopsy cases [4]. It is assumed that a high number of asymptomatic cases are undetected due to their benign behavior and slow growth [1]. With the widespread use of imaging diagnostics such as computed tomography (CT) and magnetic resonance imaging (MRI), the number of cases describing myelolipoma has increased in recent years [1].

About 15% of myelolipomas are found in extra-adrenal locations [5]. Most of them locate in presacral regions [4], although extra-adrenal myelolipomas have also been found in thorax [6], renal hilum [7], spleen [8], paravertebral regions [9], and the nasal cavity [10].

To date, presacral myelolipomas are described in fewer than 60 cases in English literature published on PubMed and form a rarity. The first patient was described by Blaisdell in 1933, concerning a case of extramedullary hematopoiesis found in a retroperitoneal tumor in an elderly woman [11].

This study aimed to systemically review and meta-analyze clinical, radiological, and epidemiological features of the presacral myelolipoma and present new case of a 71-year-old female with an incidentally detected tumor diagnosed by CT-guided biopsy.

## Methods

### Search strategy

All studies published until 30 September 2022 on the topic “presacral myelolipoma” was included in the current analysis with no restriction on age or language.

Systematic searches were performed using the term “presacral myelolipoma” on PubMed.

Articles were considered by reviewing title, abstract, and the full text if in doubt.

### Selection criteria

Inclusion criteria was a confirmed diagnosis with presacral myelolipoma. Exclusion criteria were reported cases of extra-adrenal myelolipomas that were not located in the presacral region and all research articles on the topic myelolipoma without case presentation.

The PRISMA guidelines were followed.

### Data extraction

The following information was extracted from each study: first author, year of publication, title, number of patients with presacral myelolipoma, gender, age, tumor size in diameter, reported symptoms, imaging technology used for diagnosis, and treatment.

All articles were analyzed, and a database was formed. In Table 1, all included articles are listed.

**Table 1:** List of all included case reports on presacral myelolipoma

PAT ID	Authors	Publication year	Sex	Age	Tumor size	Symptoms	Imaging	Treatment
1	Cho *et al.* [35]	2021	Female	44	14 × 11 × 8 cm	Abdominal pain	CT + MRI	Resection
2	Andriandi *et al.* [36]	2020	Female	48	1.8 × 3.3 × 1.8 cm	Atypical lower back pain	MRI	Conservative, follow-up
3	Andriandi *et al.*	2020	Female	59	4.2 × 4.2 × 4.7 cm	No symptoms	CT + MRI	Conservative, follow-up
4	Rizzo *et al.* [37]	2018	Female	72	∅ 6 cm	No symptoms	CT + MRI	Resection
5	Sethi *et al.* [38]	2018	Female	70	13 × 10 × 10 cm	Abdominal pain, urinary retention, nausea, dyspepsia	CT + MRI	Resection
6	Sakamoto *et al.* [39]	2018	Male	65	4 × 4 × 3 cm	Acute-onset abdominal pain	CT + MRI	Conservative, follow-up
7	Cho *et al.* [40]	2018	Female	70	3.5 × 3 × 3.6 cm	Pelvic pain	CT + MRI	Resection
8	Oldrini *et al.* [41]	2016	Female	65	8.5 × 7.8 cm	No symptoms	CT + MRI	Conservative, follow-up
9	Lee *et al.* [42]	2016	Female	69	∅ 7.6 cm	Abdominal pain	CT + MRI	Not mentioned
10	Lee *et al.*	2016	Female	67	∅ 4.9 cm	Urinary retention	CT + MRI	Not mentioned
11	Lee *et al.*	2016	Female	56	∅ 8.5 cm	Flatulence	CT + MRI	Not mentioned
12	Lee *et al.*	2016	Female	81	∅ 11 cm	No symptoms	CT + MRI	Not mentioned
13	Lee *et al.*	2016	Female	80	∅ 5.2 cm	No symptoms	CT + MRI	Not mentioned
14	Lazarides *et al.* [29]	2016	Female	67	6.5 × 5.5 × 2.3 cm	Numbness, weakness, and pain in lower extremities	CT + MRI	Resection
15	Tokuyama *et al.* [43]	2016	Male	71	∅ 4.3 cm	No symptoms	CT	Resection
16	Arora *et al.* [22]	2016	Male	64	5.7 × 5.2 × 4.2 cm	Lower abdominal discomfort	CT	Resection
17	Fourati *et al.* [44]	2015	Female	40	11.5 × 8.5 × 5 cm	Abdominal pain, weight loss	CT + MRI	Conservative, follow-up
18	Sagarra Cebolla *et al.* [30]	2014	Male	74	4.5 × 3.2 cm	Constipation, radiculopathy left leg	MRI	Resection
19	Varone *et al.* [45]	2014	Female	55	5 × 4 cm	No symptoms	CT	Conservative, follow-up
20	Gagliardo *et al.* [46]	2014	Female	74	Not mentioned	Lower back pain	CT + MRI	Resection
21	Itani *et al.* [46]	2014	Female	58	4.8 × 3.5 cm	Abdominal discomfort, change in bowel habits	CT + MRI	Conservative, follow-up
22	Leite MI *et al.* [47]	2014	Male	84	∅ 5 cm	Pelvic pain	CT + MRI	Resection
23	Baker *et al.* [48]	2012	Female	79	6.4 × 3.1 × 5.7 cm	No symptoms	CT + MRI	Resection
24	Asuquo *et al.* [49]	2011	Female	74	3.5 × 1.7 × 0.6 cm	No symptoms	CT	Resection
25	Spizzirri *et al.* [27]	2011	Female	69	Not mentioned	Abdominal pain, paresthesia right leg	CT + MRI	Resection
26	Gill *et al.* [13]	2010	Female	71	Not mentioned	Abdominal pain	CT + MRI	Conservative, follow-up
27	Müller *et al.* [28]	2009	Male	62	∅ 5 cm	Lower back pain	CT + MRI	Conservative, follow-up
28	Gheith *et al.* [17]	2009	Male	85	∅ 12 cm	Small bowel obstruction	CT	Resection
29	Hernández-Amate *et al.* [26]	2008	Female	64	8 × 6.5 cm	Abdominal pain, constipation, nausea, and vomiting	CT	Conservative, follow-up
30	Dann *et al.* [50]	2008	Female	82	4.5 × 3.5 cm	Abdominal pain	CT	Resection
31	Liu *et al.* [51]	2008	Female	65	11.5 × 8.5 × 5 cm	Constipation	CT	Resection
32	Skorpil *et al.* [52]	2007	Female	84	∅ 5 cm	No symptoms	CT + MRI	Resection
33	Gong *et al.* [53]	2006	Female	83	∅ 3,5 cm	Lower back pain	CT + MRI	Conservative, follow-up
34	Orsola *et al.* [18]	2005	Male	68	13 × 9 cm	Urinary retention, constipation	CT	Resection
35	Mariappan MR *et al.* [54]	2004	Male	74	10 × 8 × 5.5 cm	No symptoms	Autopsy	None
36	Giuliani *et al.* [55]	2001	Male	71	9 × 8 × 7 cm	Constipation	CT + MRI	Resection
37	Zanon *et al.* [24]	2000	Female	65	Not mentioned	Abdominal pain	Not mentioned	Resection
38	Saboorian *et al.* [56]	1999	Female	84	∅ 8.5 cm	Abdominal pain, nausea, vomiting	CT + MRI	Conservative, follow-up
39	Gavelli *et al.* [57]	1998	Female	84	15 × 12 × 10 cm	No symptoms	CT	Not mentioned
40	Adetiloye *et al.* [19]	1996	Male	1,5	Not mentioned	Urinary retention, dysuria, constipation	Ultrasound	Resection
41	Prahlow *et al.* [20]	1995	Male	68	15 × 10 × 8 cm	Lower back pain, urinary retention	CT	Resection
42	Yang *et al.* [58]	1992	Male	40	Not mentioned	No symptoms	CT	Resection
43	Grignon *et al.* [25]	1989	Female	80	∅ 12 cm	Abdominal pain	Not mentioned	Not mentioned
44	Grignon *et al.*	1989	Female	68	∅ 7 cm	No symptoms	Autopsy	None
45	Grignon *et al.*	1989	Female	83	∅ 6 cm	No symptoms	Autopsy	None
46	Chan *et al.* [23]	1988	Male	53	∅ 7 cm	Lower abdominal discomfort	CT	Resection
47	Massey *et al.* [21]	1987	Female	60	15.5 × 14.5 × 14 cm	Urinary retention	CT	Resection
48	Sutker *et al.* [59]	1985	Female	58	9 × 7.5 × 3 cm	No symptoms	CT	Resection
49	Chen *et al.* [12]	1982	Female	72	16 × 15 × 7 cm	No symptoms	Pyelography	Resection
50	Fowler *et al.* [60]	1982	Female	70	6.5 × 7 × 7 cm	Constipation, lower abdominal pain	CT	Resection
51	Labow *et al.* [61]	1977	Female	47	Not mentioned	No symptoms	Barium enema	Conservative, follow-up
52	Benson *et al.* [62]	1965	Female	52	5 × 5 × 6 cm	No symptoms	Barium enema	Resection
53	Dodge *et al.* [63]	1956	Female	74	15 × 10 × 10 cm	Abdominal pain, nausea, vomiting	Not mentioned	Conservative, follow-up
54	Blaisdell *et al.* [11]	1933	Female	64	11 × 11 cm	Cystitis, pyelonephritis	Not mentioned	Resection

### Statistical analysis

Descriptive data are presented as medians and interquartile range for non-normally distributed data, as appropriate. Categorical data are displayed as frequencies and percentages. Continuous data were tested for their normal distribution by Shapiro–Wilk analysis. Mann–Whitney *U* test was performed for continuous non-normally distributed variables. Categorical variables were compared using the chi-square test.

*p* < 0.05 was considered to indicate significance. All analyses were performed using SPSS Statistics Software version 28.0 (IBM, Armonk, New York) on macOS 12 Monterey.

## Case presentation

**Fig. 1:**
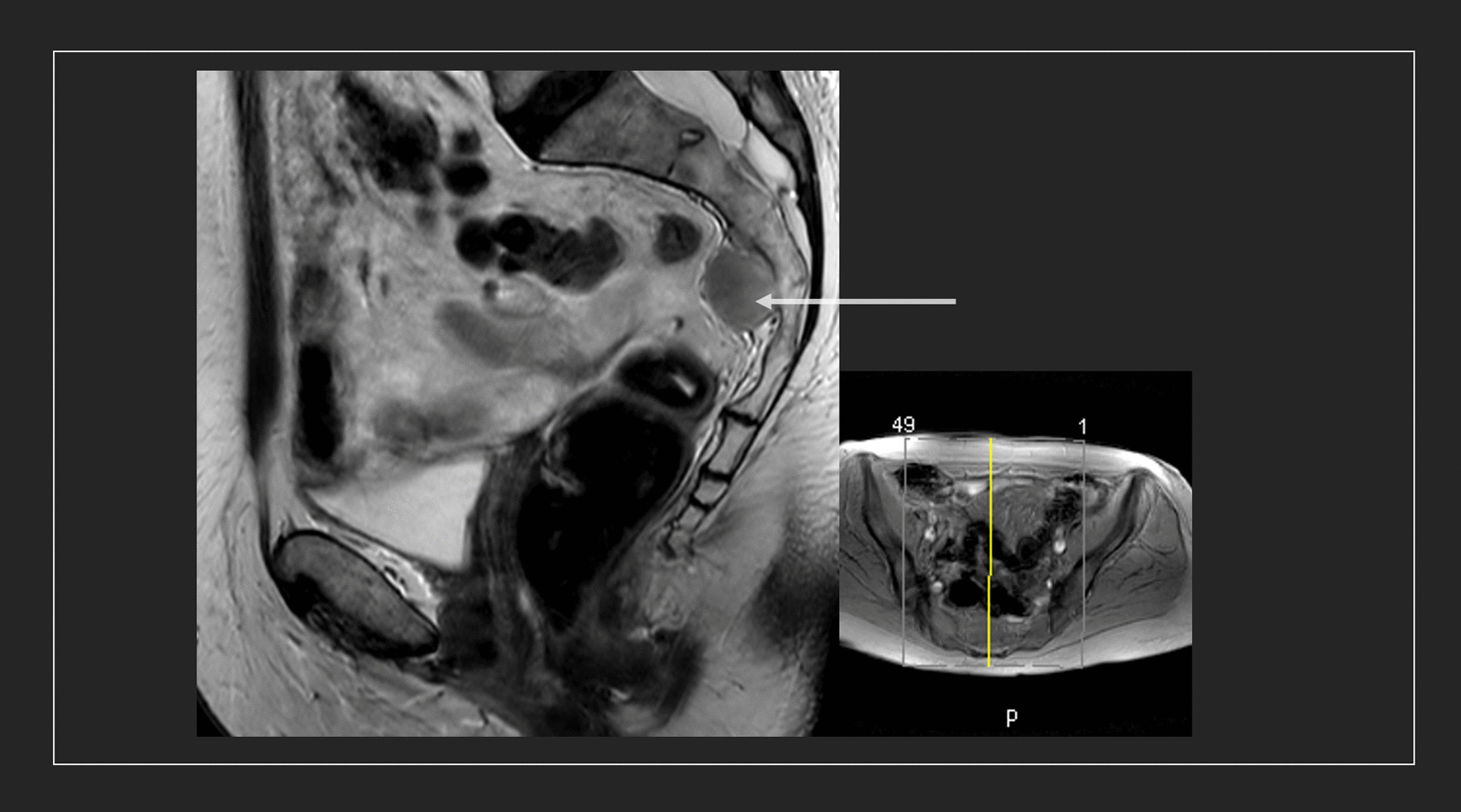
A 71-year-old female. T2-weighted (TSE) MRI scan (sagittal) of presacral myelolipoma (arrow). Image shows an incidental heterogeneous presacral tumor at the level of S3

A 71-year-old caucasian female visited our outpatient clinic with an incidentally detected retroperitoneal tumor. An exophytic presacral mass of size 2.6 × 6 × 1.9 cm (Fig. 1) was found on MRI scan, which was performed during a regular gynecological check-up. The tumor located in the soft tissue adjacent to the level of S2. The tumor was clearly demarcated and inhomogeneously configurated, which was isodense to muscle tissue. Furthermore, the tumor showed moderate contrast enhancement and infiltrated both intervertebral foramina of S2.

The patient complained of weight loss of 6 kg in 6 months without fever or diarrhea. On physical examination, no neurological deficit was found. On laboratory workup, an elevated white blood cell count (WBC) of 11.900 was found. Further analysis revealed a normal distribution of white blood cells without abnormal cell proliferation.

**Fig. 2:**
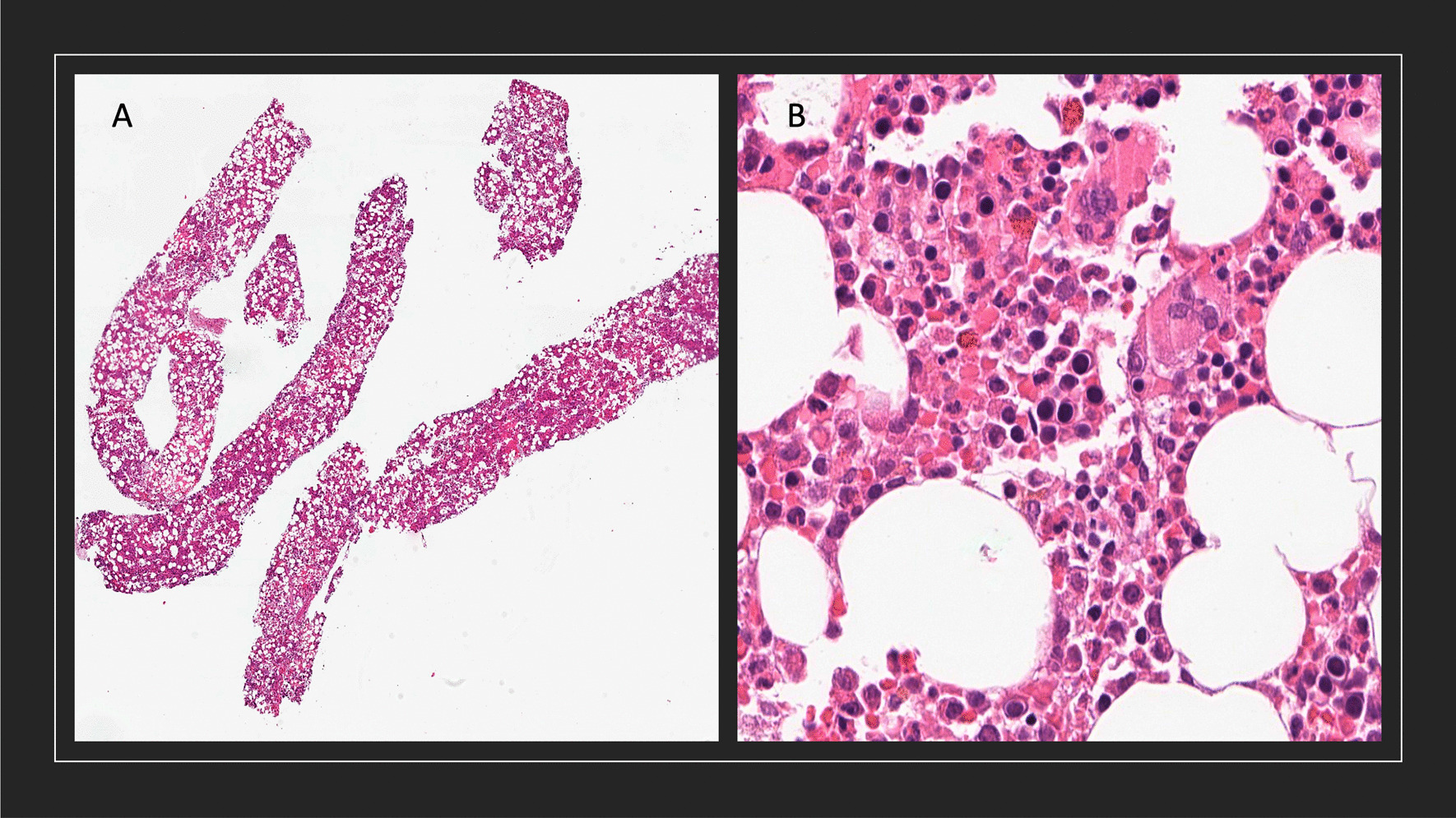
Histological image from the obtained fine-needle biopsy specimens of the presacral myelolipoma (hematoxylin and eosin staining). **A** Needle biopsy specimens (low magnification) showing monotonous histology consisting of hematopoietic cells and mature fat cells, which are intimately intermingled. No adrenal tissues are observed. **B** The hematopoietic cells show a normal trilineage from myeloid, erythroid, and megakaryocytic cells (high magnification)

A CT-guided biopsy confirmed the diagnosis of a presacral myelolipoma consisting of mature adipose cells with trilineage hematopoietic element. No ectopic adrenal tissues were observed (Fig. 2).

In consent with the patient, a decision towards further follow-up by MRI imaging of the presacral myelolipoma was given, as the patient was asymptomatic when discharged from the clinic.

At 6 months follow-up, there was no evidence of tumor enlargement by MRI imaging and the patient remained asymptomatic. Further radiological reevaluations are scheduled at 6–12-month intervals.

## Results

The PubMed database search identified 64 published articles. Through citation search, one more article was identified. Forty-seven publications with 54 reported cases were considered eligible for inclusion. Of the included articles, no longitudinal cohort studies were identified. A small number of included studies described more than one case. Lee *et al.* described five cases, whereas Grignon *et al.* and Andriandi *et al.* described three and two cases, respectively.

The earliest case included in the current analysis was from 1933, while the latest case was from 2021.

**Fig. 3:**
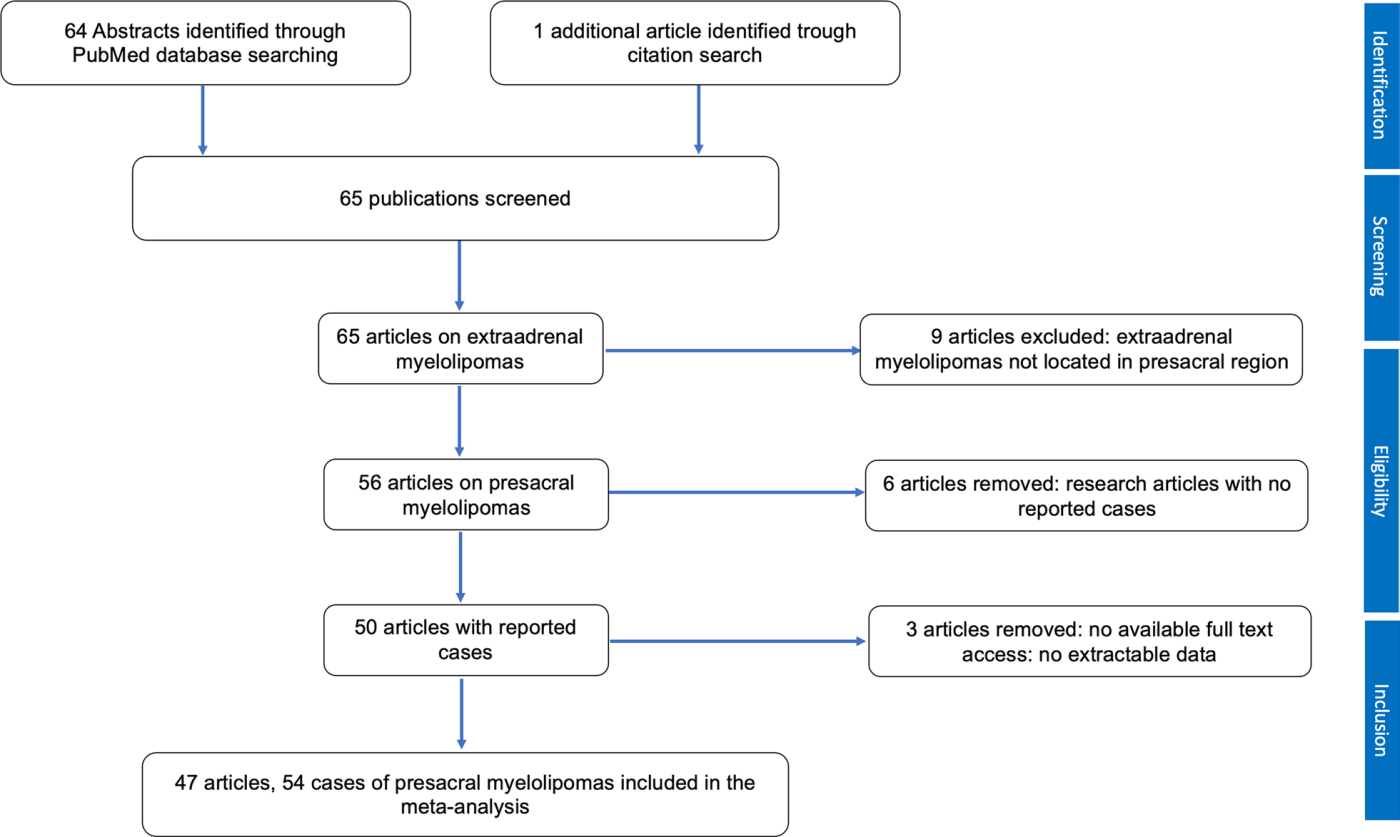
PRISMA flow diagram

Figure 3 shows the PRIMA flow diagram for included articles.

### Meta-analysis

The clinical characteristics of the 54 cases searched in literature are depicted in Table 2.

**Table 2:** Baseline characteristics between symptomatic and asymptomatic patients

	Symptomatic (*n* = 34)	Asymptomatic (*n* = 20)	*p*-Value
Age (years)	65.2 (IQR 61.5–74.0)	68.5 (IQR 58.3–79.8)	n.s.
Sex, *n* (% male)	10 (29.4%)	4 (20.0%)	n.s.
Average size (cm)	7.46 cm (IQR 4.08–11.00 cm)	6.74 cm (IQR 4.88–8.04 cm)	n.s.
Treatment, *n* (% resection)	20 (58.9%)	10 (50.0%)	n.s.

*CT* computed tomography, *MRI* magnetic resonance imaging, *IQR* interquartile range

The mean age was 66.4 years (IQR 59.7–74.0 years), ranging from 1.5 to 85 years.

Three cases were found during an autopsy. Women form the majority of reported cases, with a female predominance of 3:1.

The mean size of the presacral tumor was 7.2 cm. More than half of the patients presented with symptoms (34/54, 63%). The most frequent symptom was abdominal pain (*n* = 12), followed by abdominal discomfort (*n* = 6) and urological complaints (*n* = 6). Other symptoms presented were neurological pain in the lower extremities, lower back pain, and bowel obstruction. About half of all cases were incidentally detected (48%).

In most cases, a combination of CT and MRI was used for tumor diagnosis (46%), followed by CT only (30%). Other cases were detected by clinical and ultrasound examinations and dynamic x-ray. The diagnosis was confirmed by a fine-needle biopsy in 28 patients (52%). Thirty patients (56%) received a resection.

There was no significant difference in the size of lesions between symptomatic and asymptomatic patients (*p* = n.s.). No metastasis or recurrence was reported after an R0 resection.

## Discussion

Most presacral myelolipomas, including the one in our new case, occur in elderly female between 50 and 70 years of age [12, 13] with a mean age of 66.4 years.

### Imaging features

About half of all reported presacral myelolipomas are found incidentally on CT and MRI in the current study.

Characteristically, the yellowish mature fatty tissue within the myelolipoma appears translucent on abdominal radiographs and echogenic on ultrasound examination [5]. On CT, the fatty elements can be diagnosed by using Hounsfield units, which reveals a low attenuated tissue with −10 to −100 HU [14], while an MRI would present a high-intensity signal in T1-weighted sequence and a corresponding low-intensity signal in fat-suppressed T2 weighted sequences [5].

The hematopoietic elements of myelolipoma interspersed in mature fatty tissue usually have a medium signal intensity similar to that of the spleen on MRI [5]. On T2-weighted images, the marrow-like elements result in areas of increased signal intensity within the inhomogeneous tumor [5].

Due to its superiority in soft-tissue contrast in comparison with CT, MRI represents the modality of choice in the diagnosis of myelolipoma [15]. Thus, the potential invasion of adjacent structures, such as neuroforamina with sacral nerve compression, as described in our case report, can be detected.

In our patient, the presacral myelolipoma has indeed infiltrated neuroforamina in two distinct areas, albeit without causing any symptoms.

### Differential diagnosis and treatment recommendations

As a fat-containing soft tissue mass of the retroperitoneum, a spectrum of neoplastic conditions, such as lipoma, liposarcoma, neurogenic tumors, germ cell tumors, teratoma, and metastasis of cancer, must be considered in the differential diagnosis of myelolipoma [14].

In the current case report, Schwannoma was suspected on MRI, based on the finding of infiltration to neuroforamina. A definitive diagnosis can only be determined by fine-needle biopsy with consecutive histological examination or surgical resection. In previous cases, the diagnosis was made either in resection specimens (30 patients, 56%) or by a fine-needle biopsy (28 patients) including our patient.

According to American Association of Clinical Endocrinology (AACE)/American Association of Endocrine Surgeons (AAES) guideline from 2009 for adrenal incidentaloma, small and asymptomatic and hormonally inactive lesions < 4 cm are recommended for radiological reevaluation at 3–6 months and then annually for 1–2 years [16].

The patients may develop a variety of symptoms such as small bowel obstruction [17], urinary retention [18–21], abdominal pain and discomfort [22–26], and neurological pain [27–30], through mass effect by tumor enlargement. Tumors > 4 cm should be considered for surgical resection even without suspicion of malignancy [16].

Myelolipomas larger than 6 cm are prone to complications such as spontaneous rupture or rupture due to trivial trauma and hemorrhage with the probability of an acute abdomen [31]. Indeed, half of the patients with presacral myelolipomas, who were initially asymptomatic, later required resection of the tumor (Table 2). The biggest lesion so far reported was approximately 15 cm, described by Massey *et al.* in 1987, which caused urinary retention and azotemia associated with compression of urinary bladder neck, and the tumor was later completely resected [21].

Due to the rarity of the tumor, there are few comprehensive studies on the detailed clinical features. Han *et al.* retrospectively assessed a series of 12 patients with 13 myelolipomas in 1997 who received a conservative treatment [32] and followed up by serially conducted CT scans and reported that the tumor enlarged in 6 cases, decreased in 2 cases, and remained unchanged in 5 cases in a mean follow-up time of 3.2 years. Furthermore, most patients remained asymptomatic, and only 2 patients reported new-onset abdominal pain without life-threatening complications [32].

In our analysis, no malignant transformation or metastasis was, except for a rare infiltration secondary by other tumors (chronic lymphocytic leukemia), so far reported [17, 22].

### Etiology

The etiology of myelolipoma of the adrenal glands as well as of the presacral counterpart remains unknown. Several hypotheses have been discussed, including metaplasia of reticuloendothelial cells in blood capillaries as a response to an event such as necrosis, infection, or inflammation [32, 33]. However, nonrandom X-chromosome inactivation suggests a clonal origin of the tumor [34].

## Conclusion

Presacral myelolipoma is a rare disease with unknown etiology. In this study, we analyzed clinical, radiological features of previously reported 54 cases and reported the case of a 71-year-old woman with an incidentally detected presacral myelolipoma. For asymptomatic tumors, an observation with a regular imaging follow-up can be recommended after histological diagnosis by biopsy. Resection should be considered for bigger lesions > 4 cm and/or for symptomatic patients. Contrast-enhanced MRI and fine-needle biopsy are indicated to determine the definitive diagnosis.

## Data Availability

The dataset used in the current study is available from the corresponding author on reasonable request.

## Abbreviations

AACE: American Association of Clinical Endocrinology
AAES: American Association of Endocrine Surgeons
CT: Computed tomography
HU: Hounsfield unit
MRI: Magnetic resonance imaging
WBC: White blood cell count
